# Replacing Lectures with Small Groups: The Impact of Flipping the Residency Conference Day

**DOI:** 10.5811/westjem.2017.10.35235

**Published:** 2017-12-18

**Authors:** Andrew M. King, Chad Mayer, Michael Barrie, Sarah Greenberger, David P. Way

**Affiliations:** The Ohio State University, Wexner Medical Center, Department of Emergency Medicine, Columbus, Ohio

## Abstract

The flipped classroom, an educational alternative to the traditional lecture, has been widely adopted by educators at all levels of education and across many disciplines. In the flipped classroom, learners prepare in advance of the face-to-face meeting by learning content material on their own. Classroom time is reserved for application of the learned content to solving problems or discussing cases. Over the past year, we replaced most residency program lectures with small-group discussions using the flipped-classroom model, case-based learning, simulation and procedure labs. In the new model, residents prepared for conference by reviewing a patient case and studying suggested learning materials. Conference day was set aside for facilitated small-group discussions about the case. This is a cross-cohort study of emergency medicine residents who experienced the lecture-based curriculum to residents in the new flipped-classroom curriculum using paired comparisons (independent t-tests) on in-training exam scores while controlling for program year level. We also compared results of the evaluation of various program components. We observed no differences between cohorts on in-training examination scores. Small-group methods were rated the same across program years. Two program components in the new curriculum, an updated format of both adult and pediatric case conferences, were rated significantly higher on program quality. In preparation for didactics, residents in the new curriculum report spending more time on average with outside learning materials, including almost twice as much time reviewing textbooks. Residents found the new format of the case conferences to be of higher quality because of the inclusion of rapid-fire case discussions with targeted learning points.

## BACKGROUND

The lecture has long been the primary teaching method for graduate medical education.^1^ Because lectures can be performed with large student-to-teacher ratios, they are considered an efficient teaching method.^1^–^2^ Effectiveness of lectures as a teaching method has been called into question due to the lack of learner engagement.^2^–^3^ Lectures put the responsibility for learning in the hands of the teacher, who regulates both the sequence and depth of content coverage. Learner participation is limited to listening, taking notes, and asking clarifying questions.

Educators have sought to replace lectures with methods that promote active learning and longer term retention.^4^–^7^ One such alternative, the flipped classroom, has been widely adopted across a variety of disciplines.^7^–^10^ The premise of the flipped classroom is that learners read and study new content independently in advance of a face-to-face classroom learning session.^11^ Content is either prescribed by the instructor or independently identified by the learner and includes online learning modules, textbooks, or journal articles. Once learners are prepared, they meet with their peers in facilitated small groups to apply newly acquired knowledge to cases or problems. The flipped classroom is student-centered. Learning is driven by the learners but guided by experienced educators.^8^,^11^

Proponents of the flipped classroom hypothesize that it allows adult learners to integrate new knowledge with existing knowledge.^9^–^10^ The act of covering material at their own pace prior to a meeting promotes deeper learning, longer retention and life-long learning skills. The face-to-face classroom sessions promote knowledge application, critical thinking, and peer-faculty interactions. Additionally, flipped classrooms may prepare learners for eventual information-gathering and decision-making in complex clinical settings by mimicking real-life interprofessional interactions.^13^–^14^

Although studies of the flipped classroom are small and observational, there is growing consensus that students favor this method over the traditional lecture.^10^, ^15^–^19^ In the flipped classroom, learners use study time to build a foundation for new learning instead of spending that time reviewing lecture notes and retrofitting new knowledge with old. The flipped-classroom method not only promotes longer term retention but provides learners with cues to the depth and breadth required for use of the new knowledge in clinical application. Challenges associated with the flipped-classroom model include increased time commitment for both educators and learners, effective integration of technology, ensuring individual learner accountability, and promotion of a safe learning environment.^10^–^11^, ^18^,^20^

The Accreditation Council for Graduate Medical Education (ACGME) requires that emergency medicine (EM) residency programs provide five hours of weekly didactic instruction.^21^ Residents are required to participate in 70% of these didactics. Historically, our program has fulfilled ACGME didactic requirements through weekly lectures. For the 2015–16 academic year (AY 2016), we changed our didactic format to the flipped-classroom model.

## OBJECTIVE

The purpose of this study was to evaluate the outcomes of our first year of flipped-classroom instruction through comparison to preceding years of lecture instruction.

## CURRICULAR DESIGN

### Instructional methods

During the 2015–16 academic year, we structured our residency conference around themes covering patient presentation (e.g., chest pain, pregnancy, shortness of breath). Lectures were replaced with facilitated small-group discussions using the flipped classroom and case-based learning. Simulations and procedure sessions were also added to the conference day. Residents prepared for conference by reviewing related patient cases, and then reading recommended learning materials. Residents were also encouraged to identify and read their own learning materials. Conference time was reserved for facilitated small-group discussions about the cases, and residents were given the opportunity to apply what they learned to diagnosing and developing management plans for patient cases.

### Population

We performed a cross-sectional cohort study of EM residents who entered our program between 2011 and 2016. Our average enrollment grew over this time from n=12 per entering class to n=18. The E-2011 and E-2012 cohorts (n=28) were the last two cohorts to experience only the lecture-based curriculum. The E-2013 and E-2014 cohorts experienced both lecture-based and flipped-classroom curricula (n=31). The E-2015 and E-2016 cohorts experienced only the flipped-classroom curriculum (n=36). Our institutional review board declared this exempt research.

### Measurements

**Figure 1:**
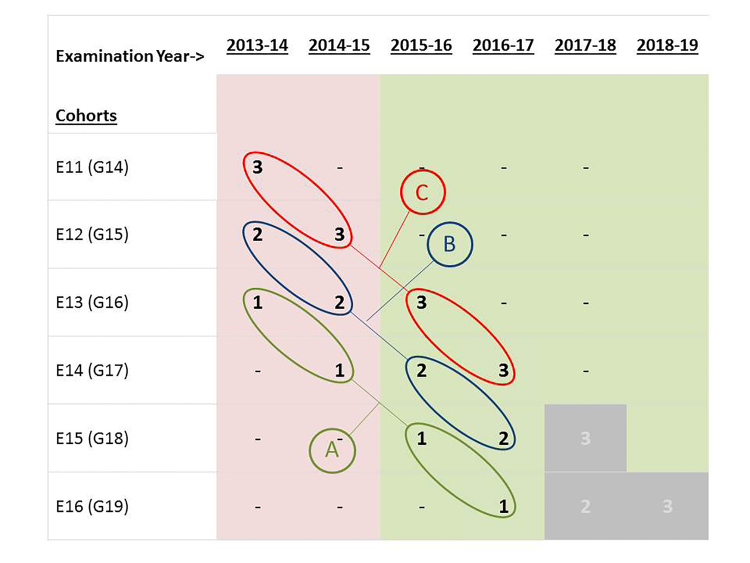
Comparison cohorts for in-training examination scores.

We compared the performance of residents who participated in the flipped classroom to those who received the lecture curriculum on the annual American Board of Emergency Medicine (ABEM) in training examination (ITE), (a standardized test normed on all residents in ACGME-accredited EM residency programs). We controlled for training level by comparing resident scores by level separately (Figure 1).

We also developed a program evaluation questionnaire to assess resident opinions of their educational experiences. The questionnaire asked residents to rate each component of the program on both quality of instruction and value to their professional development. Residents were also asked how many hours they spent with textbooks, online instruction, and journals.

### Data Analysis

While controlling for level of training (i.e., interns from the new curriculum were compared to interns in the old curriculum, etc.), we used independent t-tests to compare ITE scores for residents in the lecture-based curriculum to those in the new flipped-classroom curriculum (Figure 1).

We compared program evaluation survey results between residents who experienced the final year of the lecture curriculum (Academic Year 2014–15 [AY 2015]) to those who participated in the first year of the flipped-classroom curriculum (Academic Year 2015–16 [AY 2016]). This ensured that at least two thirds of the residents had experience with both curricula and were able to make fair comparisons. To avoid Type-1 error rates, a common problem when making multiple comparisons, we redefined the p-values considered statistically significant using the Bonferroni adjustment.^22^

## IMPACT/EFFECTIVENESS

Table 1 shows the results of the cohort comparisons on ITE scores from independent t-tests. We observed no statistical difference on the average ITE scores between residents who participated in the lecture curriculum and those from the flipped-classroom curriculum at any of the three training levels (PGY1–3).

We received program evaluation surveys from 28 of 45 residents (62.2%) in AY 2015 and from 19 of 49 residents (38.8%) in academic year AY 2016. Twenty-seven residents were eligible to participate in both surveys; however, only nine of 27 residents (33.3%) completed both.

Program component ratings of quality and value are presented in Table 2. Program components used only in one year or the other are left blank to indicate that no statistical comparison was made. Almost all program components except for *Mock Oral Boards* were rated higher in terms of both quality and value by residents in the flipped classroom. However, only two components, *adult and pediatric case conferences,* were rated significantly higher in quality, but not value (adult case conference: t=−4.0, df=45, p≤.001, es=−1.19; pediatric case conference: t=−3.7, df=45, p=.001;es=−1.10). Cohen’s d effect sizes (es) for these comparisons are considered large.^23^

*Small-group* methods were rated the same across program years. Although not significant, *lectures* were rated higher in quality and value in the flipped-classroom curriculum than they were in the lecture-based program.

**Figure 2:**
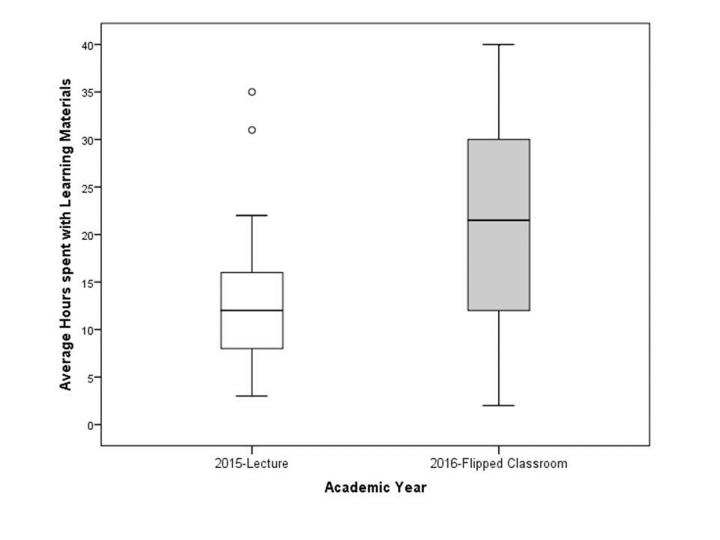
Box and whisker plot comparing average hours spent with outside learning materials such as textbooks, online learning resources and journals across two groups of residents: one from a lecture-based curriculum year (2015) and one from a flipped-classroom curriculum (2016).

Residents in the flipped-classroom curriculum reported spending significantly more hours with outside learning resources as a whole (textbooks, online learning resources, and journals combined) when compared to residents in the lecture-based curriculum (t=2.68; df=38; p=.011; es=−.852) (see Figure 2). The Cohen’s d effect size (es) for the difference in average time spent with outside learning resources (all together) is considered large.^23^ When compared separately, the amount of time spent on any one type of resource was not significantly different.

## DISCUSSION

The adoption of a flipped-classroom educational model for our large academic medical center’s EM residency program did not have any major impacts on traditional outcomes, such as standardized test results or program evaluations. Our findings are consistent with the published literature on use of the flipped-classroom model in health sciences education.^12^,^16^,^18^

Residents in the flipped classroom reported spending significantly greater amounts of time with outside learning materials: textbooks, online learning resources, and journals. This is our most significant yet not surprising finding, since preparation for small-group discussion during class meetings is a program expectation. Residents in the flipped classroom reported spending almost double the amount of time with textbooks along with roughly 25% more time with online instruction materials and journals.

Increases in time spent with preparation materials may also explain residents’ higher quality ratings of case conferences, both pediatric and adult. We believe that because residents come prepared to discuss and apply their learning to these cases that they find these activities to be of higher quality. Residents also expressed appreciation for the inclusion of rapid-fire case presentations during case conferences.

In our flipped-classroom program, the use of self-chosen learning resources was encouraged. We believe that this is appropriate at a graduate level of medical education, since preference for different types of learning resources are likely to be varied. The Free Open Access Medical Education (FOAM) movement has provided learners with a wealth of content material presented in a variety of ways from medical education experts around the world.^24^ The fact that we did not see a large, significant increase in the amount of time our residents spent with online instructional resources, is probably attributable to the fact that our lecture-group residents had also used these materials to supplement their education.

Increased use of FOAM resources combined with a flipped-classroom approach to weekly didactic sessions is helping students at the post-graduate level to customize their education,^25^ while reserving valuable group time for application of knowledge to real-world scenarios under the guidance of an expert.^26^ We expected to see higher ratings of both value and quality of most of the program components under the flipped-classroom curriculum than the lecture curriculum. However, because so few of our respondents (9 of 27) experienced both program models, we are not sure that we captured a true “curriculum change” effect. In other words, residents rated what they know, without a reference to an alternative curriculum model.

Generally, our program evaluation provided some evidence for a successful transition from a lecture-based to a flipped-classroom residency curriculum. The educational outcomes we were able to measure through standardized tests and program evaluations remained stable across the two programs. While learners in our program seemingly have responded to the flipped classroom by adopting the required preliminary learning, we are unable to confirm that the flipped-classroom model is truly superior to traditional lecture methods with regards to educational efficacy.

## LIMITATIONS

Our efforts suffer a few limitations, the worst of which was incomplete program evaluation data from our residents, particularly in the second year of the study. While we reached nearly a 40% return rate from residents in that academic year, the probability of selection bias was high. We checked for selection bias and recognize that our respondents represented more PGY-1 and 3 residents.

While the ABEM ITE assesses the collective medical knowledge of resident trainees, this single, annual assessment of medical knowledge may not be sufficiently sensitive to detect the subtle differences in educational achievement obtained from two different curriculum models. While the flipped-classroom method of teaching is designed for deeper learning and longer-term retention, an annual standardized test may not be the best measure of this type of learning.

Future studies using assessment instruments more specifically designed for measuring educational efficacy between the flipped classroom and traditional lecture methods are needed. Furthermore, study designs that are effective at isolating the type of learning that occurs in classroom didactics from the type of learning that takes place in the clinical environment could contribute to further understanding the efficacy of different curriculum methods.

## CONCLUSION

In the flipped-classroom program, residents spent more time with learning resources outside of the classroom. We see this as an indicator that they were investing more time with self-directed learning. The flipped-classroom program had no detectable effect on ITE scores and minimal effect on residents’ ratings of program components. Our findings are somewhat consistent with the findings of others. In summary, we believe that the flipped-classroom model is as educationally effective as traditional lecture methods and holds promise for further exploration. Additional studies with more sensitive assessment instruments are needed to identify potential differences in educational efficacy between the flipped classroom and traditional lecture methods.

## Figures and Tables

**Table 1:** Comparisons of ABEM in-training examination scores by cohorts of residents who participated in the flipped-classroom educational model and those who did not at different levels of training.

Cohort ->	Comparison	E-2011	E-2012	E-2013	E-2014	E-2015	E-2016	t	df	p
Level at time of test		(N=12)	(N=14)	(N=15)	(N=16)	(N=18)	(N=18)			
PGY-1	A			70.5 (6.2)		71.3 (7.6)		0.16	65	.88
								
PGY-2	B		78.2 (6.0)		75.1 (6.5)			1.93	61	.06
								
PGY-3	C	81.0 (5.5)		78.1 (5.8)				1.78	48	.08
								

*PGY*, post-graduate year; *E*, entering year; *t*, independent test value; *df*, degrees of freedom; *p*, probability value.

Comparison A: Compares first year in-training exam scores between those who experienced the flipped-classroom curriculum in year one of their residency and those who experienced a lecture-based curriculum. Comparison B: Compares second year in-training exam scores between those who experienced the flipped-classroom curriculum in year 2 of their residency and those who experienced a lecture-based curriculum in year 2 of their residency. Comparison C: Compares third year in-training exam scores between those who experience the flipped-classroom curriculum in year 3 of their residency and those who experience a lecture-based curriculum in year 3 of residency.

**Table 2:** Evaluation of program components by 28 of 45 (62.2%) residents from academic year 2015 (lecture curriculum) and 19 of 49 (38.8%) residents from academic year 2016 (flipped-classroom curriculum). Response options for quality were 1=Poor, 2=Marginal, 3=Satisfactory, 4=Good, and 5=Excellent. Response options for value were 0=No value, 1=Minimal value, 2=Moderate value, 3=Considerable value, and 4=Great value.

	AY 2015		AY 2016					
						
	Mean	SD	Mean	SD	t	df	p*	es†
Lecture: including grand rounds								
Value	3.11	.92	3.68	.89	−2.15	39.7	.037	NA
Quality	3.25	1.01	4.00	.75	−2.77	45	.008	NA
Small group								
Value	3.61	.79	3.84	.83	−0.98	37.2	.332	NA
Quality	3.56	.93	3.68	.82	−0.48	44	.631	NA
Journal club								
Value	2.68	.95						
Quality	2.38	1.06						
Procedures lab								
Value			3.95	.85				
Quality			3.63	.90				
Adult simulations								
Value			3.74	.73				
Quality			3.68	.90				
Pediatric simulation								
Value			4.11	.57				
Quality			3.89	.81				
Evidence-based medicine								
Value			2.53	.61				
Quality			2.95	.91				
Trauma M&M								
Value	3.46	.88	3.74	.81	−1.08	45	.287	NA
Quality	3.43	.92	4.05	.78	−2.42	45	.020	NA
ED M&M								
Value	3.89	.96	3.58	1.07	1.05	45	.298	NA
Quality	3.71	.90	3.95	.78	−0.92	45	.362	NA
Adult case conference								
Value	3.46	.92	4.11	.74	−2.53	45	.015	NA
Quality	3.36	.83	4.26	.65	−4.00	45	.000	−1.19
Peds case conference								
Value	3.41	.89	4.05	.780	−2.55	44	.014	NA
Quality	3.29	.85	4.16	.688	−3.70	45	.001	−1.10

*AY,* academic year; *SD,* standard deviation; *t*, independent test value; *df*, degrees of freedom; *p*, probability value; *es*, effect size; *M&M,* morbidity and mortality conference.

* Adjusted p-value for significance = .05/10 or .005

† Cohen’s D effect sizes are generally interpreted as follows: .2 = small effect, .5= medium effect, and .8=large effect.

**Table 3:** Estimates of time spent with learning materials from 22 residents in academic year 2015 (lecture-based curriculum) and 18 residents from academic year 2016 (flipped-classroom curriculum).

	AY 2015		AY 2016					
						
Time with learning materials (in hrs)	Mean	SD	Mean	SD	t	df	p*	es†
Textbooks	4.18	2.63	7.56	6.09	−2.19	22.2	.039	NA
Online instruction	7.40	6.52	9.94	6.28	−1.22	36	.230	NA
Journals	3.00	2.47	4.33	3.71	−1.34	37	.189	NA
Total time	13.77	7.96	21.83	11.04	−2.68	38	.011	−.852

*AY*, academic year, *SD,* standard deviation; *t*, independent test value; *df*, degrees of freedom; *p*, probability value; *es*, effect size.

* Adjusted p-value for significance = .05/4 or .0125

† Cohen’s D effect sizes are generally interpreted as follows: .2 = small effect, .5 = medium effect, and .8 = large effect.
